# Antimicrobial and Anti-Inflammatory Oral Effects of Fermented Lingonberry Juice—A One-Year Prospective Human Intervention Study

**DOI:** 10.1055/s-0042-1759619

**Published:** 2023-01-04

**Authors:** Pirjo Pärnänen, Sari Lomu, Ismo T. Räisänen, Taina Tervahartiala, Timo Sorsa

**Affiliations:** 1 Department of Oral and Maxillofacial Diseases, Head and Neck Center, University of Helsinki and Helsinki University Hospital, Helsinki, Finland; 2 Division of Periodontology, Department of Dental Medicine, Karolinska Institute, Huddinge, Sweden

**Keywords:** fermented lingonberry juice, caries, periodontitis, candidosis, antimicrobial, inflammation

## Abstract

**Objectives** A 1-year prospective human intervention study was performed to examine the anticaries, anti-inflammatory, antiproteolytic, and antimicrobial effects of fermented lingonberry juice (FLJ), used as a mouthwash for a period of 6 months, followed by a 6-month washout period.

**Materials and Methods** Twenty-five adults were recruited from private dental clinics in Helsinki and Joensuu (Finland). Standard oral examinations and sample gatherings were performed at base level, 6 months, and 1 year for oral *Streptococcus mutans (S. mutans)* , *Candida* , and *Lactobacilli* levels, and active matrix metalloprotease-8 (aMMP-8) levels, and for decayed, missing, filled teeth (DMFT), decayed, missing filled surfaces (DMFS) and decayed surfaces (DS) indexes, and probing pocket depths (PPDs), bleeding on probing (BOP), and visible plaque index (VPI). FLJ was used by the participants once daily for 30 seconds for 6 months. FLJ contains 0.212% (w/v) polyphenols, 3% (w/ v) sugars, and contains no excipients. Ten milliliters of FLJ were equal to 1 dL of lingonberry juice.

**Statistical Analysis** Statistical analyses were performed with nonparametric Friedman's test and pairwise post-hoc analysis with Dunn-Bonferroni test, SPSS (version 27; IBM) and *p* < 0.05 was considered as statistically significant.

**Results** The levels of *S. mutans* and *Candida* counts, DS, BOP, and VPI decreased significantly ( *p* < 0.05) during the FLJ period. *Lactobacilli* counts increased significantly, while there was also significant difference in aMMP-8 levels, DMFT, and DMFS between the three measurement points. PPDs were not affected.

**Conclusions** The specially formulated FLJ may have a positive decreasing effect on *S. mutans* , and *Candida* counts as well as decrease low-grade inflammation and proteolytic burden in the oral mucosa and periodontal tissues. The beneficial effects to the oral cavity of FLJ mouthwash may be useful among patients with oral diseases, such as dental caries, periodontitis and candidosis.

## Introduction

Dental caries and periodontal disease, two major oral infections and inflammatory diseases, affect most of the population. 1 2 In the past years, the concept that *Streptococcus mutans* ( *S. mutans* ) and *Lactobacilli* act as the keystone bacteria in caries development is under doubt, and recently a more multispecies infectious etiology has received support, 3 4 although the issue is controversial. 5 The oral normal biofilm consists of hundreds of species 6 and, e.g., the subgingival plaque related to periodontal disease has been studied to contain approximately five hundred bacterial species. The interaction of these species with each other 7 and the host have been revealed piece by piece in caries, 8 and in periodontal disease, 9 10 and that *Candida* species may have role in these diseases. 11 The idea of using *Lactobacilli* in prevention of oral microbiome dysbiosis has been suggested. 12 Studies on multispecies, and even more importantly, *in vivo* human studies revealing these interactions are still quite scarce.

To prevent dental, gingival, and mucosal infections and inflammation, additional mouthwash preparations of natural origin have been proposed. Lingonberries are low-bush wild berries, which grow in the northern hemisphere and several *in vitro* studies have shown that their phenolic substances have anti-inflammatory, 13 antimicrobial, 14 and antioxidative effects. 15 Our previous studies with fermented lingonberry juice (FLJ) specially designed for safe oral use 16 showed that it has beneficial oral antimicrobial and anti-inflammatory effects in a 2-week *in vivo* human pilot study. 17 Active matrix metalloprotease-8 (aMMP-8) chair-side point-of-care mouthrinse test has proven to be suitable to measure periodontal inflammation levels, and implemented as a biomarker in the new staging- and grading-classification of periodontal disease. 18 19 20

In this study, we aimed to evaluate the effects of 6 months use of FLJ on levels of typical oral microbes ( *Candida* , *S. mutans* , and *Lactobacilli* ), and the *in vivo* effects on hypothesized prevention of caries, inflammation, and periodontal disease.

## Materials and Methods

Twenty-five adults (aged between 28 and 91 years, mean age 64 years; M/F ratio was 10/15) were recruited randomly from private dental practices in Helsinki and Joensuu (Finland). Patients were excluded if they were pregnant, had received antibiotic medications, or if they had used chlorhexidine mouthwash during the study.

Standard oral examinations and sample gatherings were performed at the beginning of the study, and after 6 months and 1 year. The patients were instructed not to eat, drink, or brush teeth for 1 hour prior to the examinations. A saline rinse (10 mL for 30 seconds) was collected to evaluate effects on oral *S. mutans* , *Candida* , and *Lactobacilli* levels, reflecting caries and candidosis risk. Second, a chairside mouthrinse test was performed (PerioSafe, Dentognostics, Jena, Germany) according to the manufacturer's instructions to measure matrix metalloprotease-8 (aMMP-8) levels, which reflect inflammation and proteolytic periodontal destruction activity. 19 20 21 Dental decayed, missing, filled teeth (DMFT), decayed, missing, filled surfaces (DMFS) and decayed surfaces (DS) indexes were calculated and probing pocket depths (PPDs), bleeding on probing (BOP %) and visible plaque index (VPI) scale (0–3) were recorded. Saline rinse samples were cultivated by serial dilutions on Mitis salivarius (Merck, Darmstadt, Germany) bacitracin (0.2 U/mL) 20% saccharose, Sabouraud dextrose (LabM, Bury, UK) and DeMan Rogosa and Sharpe (Merck, Darmstadt, Germany) agars, and colony counts of *S. mutans* , *Candida,* and *Lactobacilli* were calculated. *S. mutans* counts were evaluated by light microscopy. CHROMagar *Candida* medium (CHROMagar, Paris, France) and latex agglutination test (Bichro-Dubli FumouzeR, Fumouze Diagnostics, Levallois-Perret, France) were used to identify *Candida* species. Scaling and root planning were performed after each sampling and oral examination time point. The participants used 10 mL of FLJ 16 (Lingora®, Vantaa, Finland) as a mouthwash for 30 second daily in addition to their normal oral homecare routines. FLJ is all natural, pasteurized, and manufactured by a patented method and contains no excipients. Naturally occurring sugars in lingonberry juice are reduced to 3g/100 mL and its polyphenol concentration is 212 mg/100 mL. Thus, the antimicrobial and anti-inflammatory effects are induced solely by the specific composition achieved by the fermentation process of lingonberry juice.

The study was conducted in accordance with the Declaration of Helsinki and approved by the Institutional Review Board (or Ethics Committee) of Stockholm Community, Sweden (2016–08–24/2016/1:8 and 2016–1-24) and the Helsinki University Central Hospital, Finland (360/13/03/00/13 and 51/13/02/2009). Informed consent was obtained from all subjects involved in the study.

Statistical analyses were performed with non-parametric Friedman's test and pairwise post-hoc analysis with Dunn-Bonferroni test (SPSS, version 27; IBM, Armonk, NY, United States), and *p* < 0.05 was considered as statistically significant. Initial correlations between the variables were calculated with Spearman's and the effects of FLJ mouthrinse with the repeated measures correlation (the rmcorr package, version 0.4.1 in R statistical software version 3.6.3). 22

## Results

A total of 21 of the 25 recruited participants used the mouthwash according to instructions (10 mL/once a day) and were included in the analyses. One patient used 5 mL/once a day, two used 10 mL irregularly, and one used 20 mL/once a day. Diseases, medications, and smoking habits are shown in Table 1 . Analysis of the microbial cultivations revealed that most of the patients' yeast species (23/25) were identified as *Candida albicans* , only two as *C. dubliniensis* . Four patients had a mixed *C. albicans* /non- *C. albicans* (nd) growth.

**Table 1:** Patient characteristics ( *n* = 21)

Age (mean ± standard deviation)	65.29 ± 16.23 years
Sex (female/ male, %)	61.9/38.1%
Smoking (yes %)	19.0%
Diseases (mean, range) 8. Medications (mean, range)	1.76, 0–4 2.95, 0–9
Medications causing xerostomia (mean, range)	1.33, 0–4

**Fig. 1:**
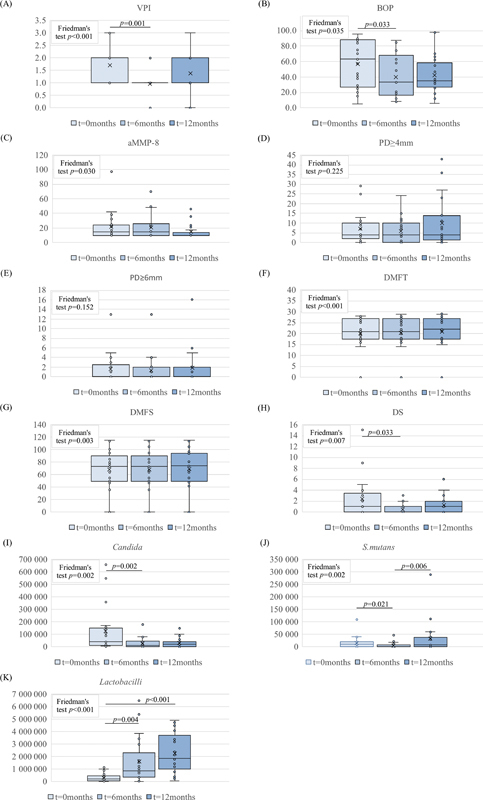
Boxplots of variables included in this study. Time points of recordings (0, 6 months, 1 year) are marked with separate colors on the x-axis. ( **A** ) Visible plaque index (VPI); ( **B** ) bleeding on probing (BOP); ( **C** ) active matrix metalloprotease (aMMP-8); ( **D** ) periodontal probing depth (PD) more than or equal to 4mm; ( **E** ) periodontal probing depth (PD) more than or equal to 6mm; ( **F** ) decayed, missing, filled teeth (DMFT); ( **G** ) decayed, missing, filled surfaces (DMFS); ( **H** ) decayed surfaces (DS); ( **I** ) *Candida* counts; ( **J** ) *Streptococcus mutans* counts; ( **K** ) lactobacilli counts. Microbial counts are reported as colony forming units/mL on the vertical axis. *p* -Values are indicated as bars above the boxplots, *p* < 0.05 was considered significant.

The effects of FLJ on the 11 parameters studied are shown in Fig. 1 . During the FLJ mouthwash period, there was a significant difference between at least two measurement points in the levels of VPI, BOP, aMMP-8, DMFT, DMFS, DS, *Candida* , *S. mutans* , and *Lactobacilli* ( *p* < 0.05). VPI ( Fig. 1A ), BOP ( Fig. 1B ), DS ( Fig. 1H ) *Candida* ( Fig. 1I ), and *S. mutans* ( Fig. 1J ) decreased significantly and *Lactobacilli* ( Fig. 1K ) increased significantly between 0 and 6 months ( *p* = 0.001, *p* = 0.033, *p* = 0.033, *p* = 0.002, *p* = 0.021, respectively). Furthermore, *S. mutans* ( Fig. 1J ) increased significantly between 6 and 12 months and *Lactobacilli* ( Fig. 1K ) increased significantly between 0 and 12 months ( *p* = 0.006 and *p* < 0.001, respectively). Finally, both aMMP-8 ( Fig. 1C ) and BOP ( Fig. 1B ) levels were smaller at 12 months compared with the beginning of FLJ (0 months), but the difference did not reach statistical significance. There were no significant differences in PD more than or equal to 4mm ( Fig. 1D ) and PD more than or equal to 6 mm ( Fig. 1E ).

Statistically significant ( *p* < 0.05) positive correlations at the beginning of the study were found between VPI versus BOP (rho = 0.700), VPI versus PD ≥ 6mm (rho = 0.491), VPI vs *S. mutans* (rho = 0.423), BOP vs PD≥ 6mm (rho = 0.418), aMMP-8 versus *Candida* counts (rho = 0.502), aMMP-8 vs *Lactobacilli* counts (rho = 0.397), DMFT versus DMFS (rho = 0.818), PD≥ 4 versus PD ≥ 6mm (rho = 0.461), PD ≥ 6mm versus *Candida* counts (rho = 0.413), *Candida* versus *Lactobacilli* counts (rho = 0.570), and *S. mutans* versus *Lactobacilli* counts (rho = 0.399). During the FLJ mouthwash period, significant repeated measures correlations ( *p* < 0.05) were found between *Candida* counts versus DS (rmcorr = 0.572), *Lactobacilli* counts versus VPI (rmcorr = -0.380), and BOP versus VPI (rmcorr = 0.383). Importantly, nearly significant correlations were found between *Lactobacilli* versus DMFT (rmcorr = 0.241; *p* = 0.120), *Lactobacilli* counts versus BOP (rmcorr = -0.291, *p* = 0.058), and *Lactobacilli* versus *Candida* (rmcorr = 0.287; *p* = 0.062).

## Discussion

The search for the pathogen(s) causing caries has been ongoing for decades and most studies indicate that this multifactorial disease incidence increases due to high carbohydrate diet frequency and the presence of acidogenic microbes (not necessarily *S. mutans* ), failing of salivary protective properties, vulnerable tooth anatomy, or insufficient manual cleaning of the teeth, resulting in enamel and dentin decay. The main aim is to prevent disease initiation and eventual progress by aiding host defensive mechanisms. The protection of teeth with fluoride has been the most effective established preventive measure against caries. In symbiotic conditions, microbial balance is maintained by host–microbial and microbial–microbial interactions, and even opportunistic pathogens exist side by side. When this balance is disturbed depending on, for example, host immune deficiencies, local excess of carbohydrates, mechanical trauma, or deepened gingival probing depths, increased anaerobic conditions create optimal environments for the dysbiotic and opportunistic pathogen proliferation or activation of specific destructive enzymatic production and cascades. 4 20 23 24 25 The basic building blocks of dentin and periodontium are proteins, and collagens are found in both structures. Minute protein structural difference and the specificity of each microbial proteolytic enzyme may be the determinant by which species will flourish. Several oral microbes, such as *Candida* , *S. mutans* , and *Porphyromonas gingivalis* , possess collagenolytic or basement membrane degrading proteolytic enzymes or have proteins that interact with the host to enhance adhesion and subsequent invasion. 26 On the species level, it is about survival of the fittest, a continuous race for nutrients, living space and beat the host defense mechanisms. According to the current understanding, a symbiotic oral microbiota to maintain or restore oral health is prevalent. 27

In this study, FLJ juice caused statistically significant reductions in *Candida* and *S. mutans* levels, and *Candida* levels remained at a lower level at the 1-year time point. Also, caries incidence (DS) decreased significantly during the mouthwash period and slowly reverted to near study onset level. May *Candida* yeasts play a more pivotal role in caries? They are also acidogenic and some evidence points to that direction. 28 The caries indexes DMFT/DMFS/DS gave a dissimilar picture of the caries incidence. This is due to the fact that secondary caries findings are concealed if they occur on the same tooth surface as missing or filled surfaces; the same surface is counted only once. Therefore, the authors state that DS gives a more accurate picture of caries incidence. High *S. mutans* levels have also been found to be coassociated with severe untreated periodontitis. 29 *Lactobacilli* counts rose significantly in this study during the FLJ period. *Lactobacilli* are associated and found from carious lesions, and they are acidogenic, but according to this study, no significant correlation was found with caries incidence (DS) and surprisingly, negative correlation with VPI. *Lactobacilli* has not shown significant upregulation of gene expressions of collagenolytic enzymes in root caries. 30 The advantage in increasing the patient's own *Lactobacilli* counts is that the effect remained even after the washout period, compared with commercial probiotic *Lactobacilli* preparations whose colonization is only very short-term. *Lactobacilli* are proposed to inhibit *Candida* by competing with niches and adhesion, production of lactic or organic acids, inhibiting inflammation or altering gene expression, 31 and may be beneficial in chemotherapy-related mucositis. 32 *Candida* and streptococci may have mutualistic interactions in the oral cavity increasing binding or increase drug resistance, as opposed to antagonistic effects of *Lactobacilli* against *Candida* . 7

BOP and VPI decreased significantly by FLJ. At the same time, aMMP-8 levels were decreasing that may be indicative of diminished inflammatory and tissue destructive proteolytic burden. The basic metabolism of gingival tissue is based on continuous tissue component remodeling of proteins, but excessive proteolytic and collagenolytic inflammation eventually may cause irreversible tissue damage. *Candida* yeasts have been proven to be gelatinolytic and *Candida glabrata* cell wall proteases are able to convert pro-MMP-8 to its active forms *in vitro* , and this activation was inhibited by FLJ. 21 The chair-side point-of-care aMMP-8 test is a valuable tool to diagnose and follow treatment efficacy and is more precise than BOP in staging and grading of periodontal disease. 19 20 As the key pathogens in periodontitis are not known, scaling and root planning and oral homecare instructing have been the best treatment options. Additional chlorhexidine is applied as adjunctive therapy in difficult cases. Chlorhexidine is a broad-spectrum antiseptic but has multiple side effects and cannot be used for prolonged periods, 33 and there is inconclusive evidence of its effect on lowering *S. mutans* levels. 34 35 By contrast FLJ does not inhibit the growth of *Lactobacilli in vitro* or *in vivo* , and none of the participants in the current and previous studies 16 17 reported any side effects. Lingonberries are known to have antimicrobial, anti-inflammatory, and antioxidant effects. 36 Clinical human trials have shown beneficial effects of FLJ on salivary parameters and reducing xerostomia, 37 and potential inhibition of plaque levels, bleeding on probing, and inflammation in dental implants. 38

## Conclusions

This is the first *in vivo* prospective study to our knowledge of the effects of lingonberries in the oral environment. FLJ seems to offer a safe addition to oral homecare, potentially decreasing visible plaque, BOP, *Candida* , and *S. mutans* levels, caries risk as well as periodontal low-grade inflammation and proteolytic tissue destructive aMMP-8 burden *in vivo* without side effects. The search for identifying culprit microbes causing these diseases is still the ultimate target, and further randomized double-blinded placebo-controlled *in vivo* studies are required to verify these results.
